# QSOX2 Is an E2F1 Target Gene and a Novel Serum Biomarker for Monitoring Tumor Growth and Predicting Survival in Advanced NSCLC

**DOI:** 10.3389/fcell.2021.688798

**Published:** 2021-07-19

**Authors:** Yaqi Li, Mei Liu, Zhuoxian Zhang, Libin Deng, ZhenYu Zhai, Hua Liu, Yiting Wang, Cheng Zhang, Jianping Xiong, Chao Shi

**Affiliations:** ^1^Department of Oncology, The First Affiliated Hospital of Nanchang University, Nanchang, China; ^2^Institute of Translational Medicine, Nanchang University, Nanchang, China; ^3^Center for Experimental Medicine, The First Affiliated Hospital of Nanchang University, Nanchang, China; ^4^Department of Clinical Laboratory, The Fourth Affiliated Hospital, Nanchang University, Nanchang, China; ^5^School of Basic Medical Sciences, Nanchang University, Nanchang, China

**Keywords:** quiescin Q6 sulfhydryl oxidase 2, E2F transcription factor 1, non-small cell lung cancer, cell cycle, biomarker

## Abstract

**Background:**

Quiescin Q6 sulfhydryl oxidase 2 (QSOX2), an enzyme that can be directly secreted into the extracellular space, is known to be associated with oxidative protein folding. However, whether *QSOX2* is abnormally expressed in non-small cell lung cancer (NSCLC) and its role in tumor growth remains unclear.

**Methods:**

Real-time quantitative PCR (qPCR), immunohistochemistry (IHC), bioinformatics analyses were applied to analyze the expression pattern and prognostic significance of QSOX2 in NSCLC. Xenografts model, enzyme-linked immunosorbent assays (ELISA), western blot analysis (WB), and IHC were preformed to examine *in vivo* tumor suppression and intracellular and extracellular expression of QSOX2. Flow cytometry, WB and qPCR analyses were used to elucidate the role of QSOX2 in cell cycle regulation. Chromatin immunoprecipitation assay (ChIP) assay and Dual-Luciferase reporter assay were employed to investigate transcriptional regulation of *QSOX2* by E2F Transcription Factor 1 (*E2F1*).

**Results:**

Quiescin sulfhydryl oxidase 2 was significantly overexpressed in NSCLC and associated with poor survival in advanced-stage patients. The intracellular and extracellular expression of QSOX2 by tumor cells markedly decreased after anti-cancer therapy *in vitro*, *in vivo* and in the clinic. Moreover, *QSOX2* silencing in NSCLC cell lines resulted in inhibition of cancer cell proliferation, induction of apoptosis, and decreased expression of cell division-related genes (CENPF and NUSAP1) and Wnt pathway activators (PRRX2 and Nuc-β-catenin). Mechanistically, QSOX2 was expressed periodically during cell cycle and directly regulated by E2F1.

**Conclusions:**

Our findings demonstrate that QSOX2 is directly regulated by E2F1 in the cell cycle, which is essential for the proliferation of NSCLC cells. Furthermore, QSOX2 is a prognostic indicator for NSCLC and may be developed into a biomarker for monitoring tumor burden and therapeutic progress.

## Introduction

Lung cancer has the highest incidence and death rates worldwide. Although the trends for the incidence rates of lung and bronchial cancers are decreasing, the death rates are still the highest among all cancers (Siegel et al., 2018). Non-small cell lung cancer (NSCLC), the most common subtype of lung cancer, accounts for more than 80% of lung cancer cases (Ettinger et al., 2019). Multiple biomarkers, such as carcinoembryonic antigen (CEA), cancer antigen (CA)-199 and cytokeratin-19 fragment antigen 21-1 (CYFRA21-1), have been used in the clinic as markers to monitor the response of NSCLC patients receiving antitumor treatment. However, traditional serum markers have poor specificity in monitoring tumor cell cycle activity after anticancer treatment due to the bias of their pathological type and exocrine state in NSCLC (Hu et al., 2016). Therefore, it is necessary to identify biomarkers that are convenient to assess and can specifically reflect changes in tumor proliferation after anticancer treatment in advanced NSCLC.

The quiescin sulfhydryl oxidase (QSOX) family includes flavoproteins that catalyze the insertion of disulfide bonds into unfolded reduced proteins with a concomitant reduction of oxygen into hydrogen peroxide within the intracellular or extracellular space (Raje and Thorpe, 2003). Importantly, the formation of disulfide bonds is required for protein folding, protein stability, protein function and linkage of different proteins (Sevier et al., 2001; Morel et al., 2007; Ilani et al., 2013). In terms of categories, two genes, quiescin sulfhydryl oxidase 1 (*QSOX1*) and quiescin sulfhydryl oxidase 2 (*QSOX2*), encode QSOX proteins, and each *QSOX* gene generates both a long and a short transcript due to alternative splicing (Radom et al., 2006; Kuo et al., 2017). Previous studies have confirmed that QSOX1 activity can modulate the architecture and properties of the extracellular matrix (ECM) and protect against oxidative stress-induced cell apoptosis in several malignancies (Ilani et al., 2013; Lake and Faigel, 2014; Morel et al., 2007), and recent studies have shown that QSOX1 is associated with metastasis or progression in lung cancer (Sung et al., 2018), breast cancer (Knutsvik et al., 2016), prostate cancer (Baek et al., 2018), and pancreatic ductal adenocarcinoma (Hanavan et al., 2015), suggesting that *QSOX1* is one of the critical genes in tumor progression. In total, 41.2% of the primary structure and all of the key features of the *QSOX2* sequence are shared with the *QSOX1* sequence (Wittke et al., 2003; Coppock and Thorpe, 2006). Pei-Shiue Jason Tsai and coworkers found that QSOX2 had a distinct association with the sperm acrosome and implantation fossa during different stages of epididymal maturation (Wang et al., 2018). However, only one published report has found that the overexpression of *QSOX2* increases the sensitivity of neuroblastoma cells to proapoptotic stimuli. Whether the expression of *QSOX2* plays a regulatory role in tumor growth should be further studied.

Accelerated cell cycling promote cancer cell proliferation, and regulatory transcription factors are required for the initiation and progression of the cell cycle. Numerous studies have confirmed that the adenoviral early region 2 binding factor (E2F) family is tightly regulated throughout the cell cycle via transcriptional and translational regulation, and heightened oncogenic E2F activity in virtually all cancers results in uncontrolled cell proliferation (Kent and Leone, 2019). E2F Transcription Factor 1 (E2F1) is the first member of the E2F family. During cell cycle progression, cyclin-dependent kinases (CDKs) phosphorylate pRB, releasing E2F1, which then transactivates and increases the levels of downstream target genes during G1 in order for cells to pass the restriction point and progress to S phase (Kwon et al., 2017). Recent studies have demonstrated that E2F1 plays pivotal roles in regulating tumor proliferation and apoptosis via regulating the p53 pathway (Engelmann and Putzer, 2012; Kent and Leone, 2019). Upstream, oncogene-driven E2F activity accelerates the cell cycle and induces aberrant cell proliferation (Kent and Leone, 2019). Downstream, many E2F target genes are involved in mitosis, chromosome instability (CIN) and aberrant genome duplication (Sotillo et al., 2010; Vaidyanathan et al., 2016). Moreover, in addition to exerting cell cycle control, E2F1 regulates the expression of a class of genes critical for angiogenesis, interactions between tumor cells and mesenchymal cells and ECM remodeling to promote invasion and metastasis (Hollern et al., 2014). These findings suggest that the mechanisms by which E2F1 regulates the proliferation, apoptosis, invasion and metastasis of tumor cells are complex. Additional functional genes involved in tumor progression regulated by E2F1 need to be further identified.

In this study, we found that *QSOX2* expression was upregulated in NSCLC and that *QSOX2* overexpression indicated a poor prognosis in NSCLC. Our results also suggest that *QSOX2* can serve as a specific circulating marker for monitoring cell proliferation after anticancer therapy in NSCLC. Mechanistically, we identified *QSOX2* as a cell cycle-related gene transcriptionally regulated by *E2F1* in G1 phase and showed that *QSOX2* silencing could significantly inhibit tumor growth, promote apoptosis and downregulate the expression of cell division-related proteins [centromere protein F (CENPF) and nucleolar and spindle associated protein 1 (NUSAP1)] and Wnt pathway activators [paired mesoderm homeobox protein 2 (PRRX3) and Nuc-β-catenin].

## Materials and Methods

### Patients and Specimens

For cohort 1, samples of NSCLC tissue and adjacent tissue were collected from 22 patients who had undergone curative surgery between 2017 and 2018 at The First Affiliated Hospital of Nanchang University (Supplementary Table 1A). The mRNA from frozen tissue samples was subjected to reverse-transcription quantitative real-time PCR (RT-qPCR) analysis. Serum samples were collected from the 22 patients before surgery and 22 healthy persons and subjected to enzyme-linked immunosorbent assay (ELISA) analysis. For cohort 2, samples of NSCLC tissue and adjacent tissue were collected from 34 patients who underwent curative surgery between 2016 and 2018 at The First Affiliated Hospital of Nanchang University (Supplementary Table 1B). Formalin-embedded tissue sections (without follow-up data) were subjected to immunohistochemistry (IHC). The samples in cohorts 1 and 2 were reviewed by a pathologist to confirm the diagnosis of NSCLC. For cohort 3, tissue microarrays (TMAs) containing 93 pairs of NSCLC tissue and matched normal tissue with complete clinicopathological information and follow-up data^1^ were purchased from Shanghai Outdo Biotech (HLugA180Su03). For cohort 4, TMAs containing 28 pairs of NSCLC tissue and matched normal tissue with complete clinicopathological information were purchased from Wuhan Iwill Biotech (IWLT-N-70L43) (Supplementary Table 1C). For cohort 5, Serum samples were collected from the 17 patients before and after 2 cycle of chemotherapy and subjected to ELISA analysis (Supplementary Table 1D). The study methodologies conformed to the standards set by the Declaration of Helsinki. This study was approved by the Institutional Ethics Committee of the First Affiliated Hospital of Nanchang University.

### Bioinformatic Analysis

Publicly available datasets with gene expression profiles of NSCLC and normal tissues and corresponding clinicopathological and survival information were identified and downloaded from the gene expression omnibus (GEO) (GSE75037) and The Cancer Genome Atlas (TCGA)^2^ databases. A gene expression array dataset of primary lung cancer cell lines was downloaded from GSE32036. A gene expression array data set of double thymidine block-released U2OS cells was downloaded from GSE52100. Bioinformatic computations and figure drawing were performed with several packages (limma, survival, ggplot2, fgsea, and circlize) in the statistical software package R, version 3.6.1^3^, and WEB-based GEne SeT AnaLysis Toolkit^4^.

### Cell Culture and Transfection

Human lung cancer cell lines (NCI-H1299, NCI-H3255, and NCI-H1975) were obtained from American Type Culture Collection (ATCC). The cells were cultured in Roswell Park Memorial Institute (RPMI)-1640 medium (Gibco, Thermo Fisher Scientific, United States) supplemented with 10% fetal bovine serum (FBS; BI, Biological Industries) in a humidified incubator with 5% CO_2_ at 37°C. Small interfering RNAs (siRNAs) against *QSOX2* and *E2F1* and a scrambled siRNA negative control were synthesized by GenePharma. The siRNAs were diluted to a working concentration of 20 nM using DEPC-treated water according to the manufacturer’s guidelines. All siRNAs were transfected using TurboFect Transfection Reagent (Thermo Scientific, Inc.). All the siRNA sequences are reported in Supplementary Table 2. Transfection efficiency was detected by fluorescence microscopy and then verified by Western blot (WB) analysis.

### Western Blotting and RT-qPCR

Total soluble proteins were prepared by lysis in RIPA buffer containing protein inhibitor and phosphatase inhibitor cocktails. The treated-cell lysates were collected for Western blotting, subjected to 10% SDS-PAGE and transferred electrophoretically to PVDF membranes. The blots were incubated with a specific primary antibody against QSOX2 (1:1,000; cat no. ab121376; Abcam), E2F1 (1:500; cat no. ab179445; Abcam), cyclin B1 (CCNB1) (1:500; cat no. A2056; ABclonal), NUSAP1 (1:1,000; cat no. abs134563; Absin), cyclin E2 (CCNE2) (1:500; cat no. A4272; ABclonal), PRRX2 (1:500; cat no. 23869-1-AP, Proteintech), Histone H3 (1:1000; cat no. abs131870, Absin), β-catenin (1:500; cat no. 610154, BD) or glyceraldehyde 3-phosphate dehydrogenase (GAPDH) (1:1,000; cat no. HC301-01, TransGen Biotech), and then the appropriate secondary antibody (a goat anti-rabbit or anti-mouse HRP-conjugated antibody) was added. The target proteins were visualized by using a chemiluminescence reagent (Thermo Fisher Scientific). As an exception, CENPF (1:1,000; cat no. Ab90; Abcam) was evaluated using 6% SDS-PAGE owing to the 367-kDa molecular weight of the protein.

For real-time PCR, total RNA was isolated from tissue and then converted into cDNA using a reverse transcription reagent kit (Applied Biosystems, Foster City, CA, United States). RNA expression was measured with SYBR Green qPCR Master Mix (Code DRR081A, Takara) on an ABI step plus one sequence detection system (Applied Biosystems). The PCR conditions was set as: 95°C for 5 min; 40 cycles of 95°C for 15 s, 60°C for 1 min and 72°C for 30 s. GAPDH was confirmed as an internal reference by GeNorm data (Supplementary File 1) follows the Minimum Information for Publication of Quantitative Real-Time PCR Experiments (MIQE) guidelines. The relative expression levels were calculated using the 2^–ΔΔ*Ct*^ method. Experiments were performed three times. All specific primers used for PCR amplification are listed in Supplementary Table 3.

### Cell Cycle Synchronization and Analysis

293T cells were synchronized using a double thymidine block protocol before cell cycle analysis. The adherent cells were treated with 2 mM thymidine for 17 h at <50% confluence. Fresh medium was added after the first block for 8 h. The cells were again exposed to 2 mM thymidine for 15 h. After the second block, the cells were cultured with fresh medium for 0, 2, 4, 6, 8, or 10 h. The cells and cell lysates were collected to undergo cell cycle and WB analyses. For cell cycle analysis, cells were harvested and fixed in 1% paraformaldehyde for 45 min and 70% ethanol for 4 h at 4°C. The cells were then suspended in 1 ml of phosphate-buffered saline (PBS) containing propidium iodide (50 μg/ml) and RNase (50 μg/ml) for 30 min at 37°C in the dark. The cell cycle distribution was analyzed by a flow cytometer, and the data were analyzed using Kaluza 1.3 software (Beckman Coulter).

### Apoptosis Assay

Transfected NSCLC cells were grown to 70–80% confluence and subjected to an apoptosis assay. Cell suspensions were harvested, dissociated enzymatically into single cells with trypsin and then treated with the FITC Annexin V apoptosis detection kit I (BD Biosciences Pharmingen) according to the manufacturer’s protocol. The samples were immediately analyzed by flow cytometry (BD FACS Canto II, United States). The results were evaluated with FlowJo V10 software. The dots in the lower left quadrant, upper left quadrant and upper right quadrant represent the viable cells, the cells in early apoptosis and the cells in late apoptosis, respectively.

### Cell Proliferation and EdU Assays

Non-small cell lung cancer cells were transfected with the indicated siRNAs, and the proliferation rates from 12 to 120 h were measured using flow cytometry. For 5-ethynyl-2-deoxyuridine (EdU) assays, the transfected cells were resuspended and then placed in 96-well plates. For observation of cell proliferation, EdU was used to detect cell DNA replication activity by integrating with the Apollo fluorescent dye. The EdU kit applied in the experiment was purchased from RiboBio (RiboBio Co., Ltd., Guangzhou, China). The procedures were performed according to the manufacturer’s instructions, and the results were observed under a fluorescence microscope.

### IHC

Formalin-fixed, paraffin-embedded (FFPE) tissues from human NSCLC samples (cohort 2) and NCI-H1299 xenografts were cut into 4-μm-thick sections and mounted onto slides. TMA cohort 3 was utilized to determine the connection between QSOX2 and the prognosis of NSCLC patients, and TMA cohort 4 was evaluated to verify the relationship between QSOX2 and E2F1. These slices and TMAs were subjected to IHC using the two-step method of SPlink Detection Kits (Zhongshan Biotechnology, China), and immunoreactivity was visualized using a Polink-2 HRP DAB Detection kit (Zhongshan Biotechnology, China) according to the manufacturer’s procedure. An FSX100 microscope equipped with a digital camera system (Olympus) was used to obtain IHC images. The tumor tissues incubated without primary antibody was used as a negative control. The IHC score was computed via the German semiquantitative scoring method (Remmele and Schicketanz, 1993). Each slide was scored for the intensity of staining (no staining = 0; weak staining = 1; moderate staining = 2; and strong staining = 3) and the percentage of positive cells (0% = 0; 1–24% = 1; 25–49% = 2; 50–74% = 3; and 75–100% = 4). The final immunoreactive score was determined using the following formula: Total score = intensity score multiplied by the percentage score. The score was assessed independently by two proficient pathologists. For statistical analysis, the immunoreactive scores of the samples in cohort 3 were grouped into two categories, with scores of 0–6 being considered negative/low expression and scores >6–12 being considered median/high expression.

### Chromatin Immunoprecipitation (ChIP) Assay

Cells were fixed and cross-linked at 37°C for 10 min with 1% formaldehyde and gently swirled to mix. The cross-linked cells were resuspended in 300 μl of chromatin immunoprecipitation (ChIP) lysis buffer and mixed at 4°C. Then, sonication was performed at level 2 (Ultrasonic Processor, Sonics) for 30 s to yield fragments of 100–400 bp, and an immunoprecipitating antibody (anti-E2F1) was added to the supernatant fractions. The eluted DNA was recovered with QIAquick columns (Qiagen) and used as a template for PCR amplification. The predicted binding sequences and the primers used for the QSOX2 promoter are listed in Supplementary Table 3. The specificity of each primer for the promoter region of the *QSOX2* gene was examined by sequencing after amplication (Data not shown).

### Dual-Luciferase Reporter Assay

The predicted E2F1 transcription factor binding site in the 5′-upstream region (−1000 ∼ +1) of the human *QSOX2* gene was analyzed using JASPAR software^5^. For construction of a reporter vector for a luciferase assay, the 5′-fragment of the human *QSOX2* gene containing E2F1 binding sites (−500) was amplified by genomic PCR and cloned into the firefly luciferase reporter plasmid pGL3.0 (Promega, Madison, WI, United States). Then, 293T cells were seeded in a 12-well plate, followed by co-transfection of 0.2 μg/well wild-type or mutated pGL3.0-Basic-QSOX2 promoter-containing reporter plasmids, siRNAs-E2F1 and 5 ng/well of the internal control plasmid pRL-TK. Twenty-four hours after transfection, the cells were harvested to assess luciferase activity using a Dual-Glo Luciferase system (Promega) according to the manufacturer’s protocol. All experiments were performed in triplicate. The inserted sequences are listed in Supplementary Table 4.

### Tumor Xenografts

For *in vivo* experiments, 0.5 × 10^7^ NCI-H1299 cells were resuspended in sterile PBS (200 μl) and injected subcutaneously into both flanks of 5-week-old female BALB/c-nu mice (SLAC Laboratory Animal Co., Ltd., Hunan, China). Six female nude mice (4–5 weeks old) were included in each group. Tumor growth was monitored and measured with calipers every 3 days. The xenografts were allowed to grow for 18 days, up to the formation of palpable tumors. Control animals received intraperitoneal (i.p.) injections of 0.9% NaCl once every 3 days, while the therapeutic group received i.p. injections of 1 mg/kg cisplatin (Qilu Pharmaceutical, China) once every 3 days for a total of three injections per mouse. The xenografts were harvested for immunohistochemical and WB analyses. The change in the QSOX2 concentration in the serum per mouse was detected by ELISA (cat no. JL48206; Shanghai Jianglai Biotechnology, China). Animal experiments were approved by the Institutional Animal Care and Use Committee of the First Affiliated Hospital of Nanchang University.

### Statistical Analysis

Differences in quantitative data between two groups were estimated using a 2-sided paired or unpaired Student’s *t*-test. The intraclass correlation coefficients for the IHC scores of two proteins were analyzed by the Spearman correlation coefficient. The prognostic value of *QSOX2* was analyzed by Kaplan-Meier analysis. Specific comparison of IHC scores between two independent groups was performed using the Mann Whitney *U* test. The χ^2^ test was used to analyze the correlation of gene expression and clinicopathological characteristics. Receiver operating characteristic (ROS) was performed to assess the diagnostic potential of *QSOX2* in NSCLC. All of the analyses were performed using SPSS software version 18.0 (SPSS, Chicago, IL, United States). For all statistical methods, *P* < 0.05 was considered significant.

## Results

### *QSOX2* Expression Is Abnormally Elevated in NSCLC Tissue and Predicts a Poor Clinical Outcome

**FIGURE 1:**
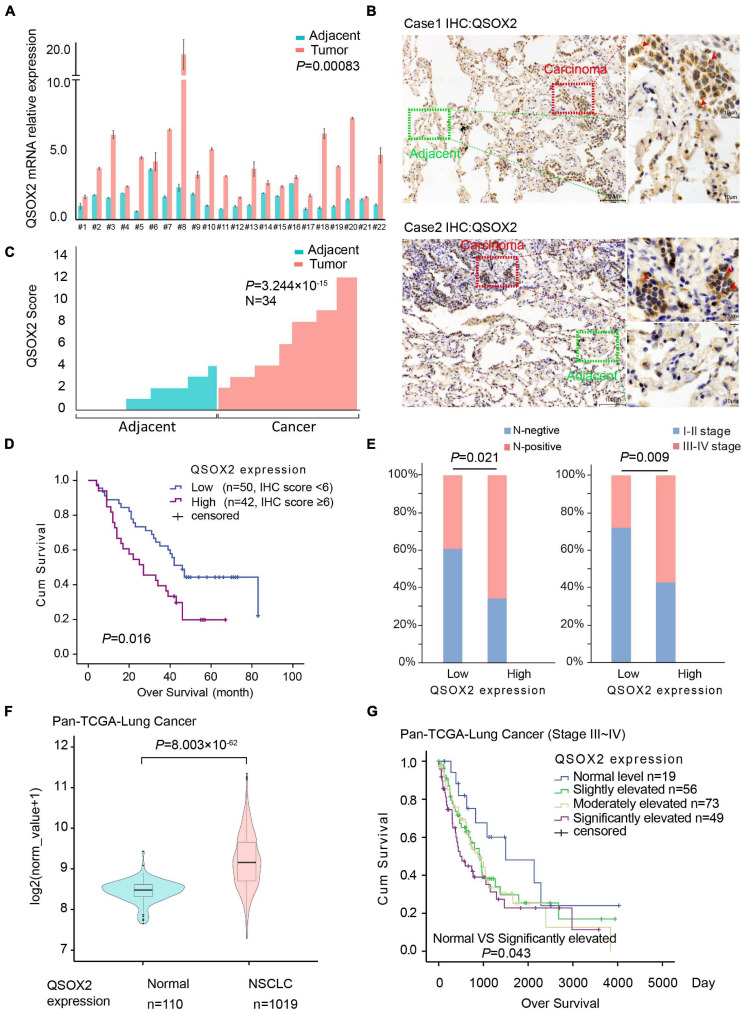
Quiescin sulfhydryl oxidase 2 (*QSOX2*) is highly expressed in NSCLC and correlates with prognosis. **(A)** Twenty-two pairs of resected frozen tissue samples were subjected to real-time PCR analysis for *QSOX2* expression (cohort 1). **(B)** IHC detection of QSOX2 expression in representative NSCLC samples and matched adjacent normal lung tissue samples (*n* = 34, cohort 2). The areas of carcinoma and adjacent tissues are indicated. The red arrow head represents QSOX2. **(C)** The expression of QSOX2 according to the IHC score. Statistical significance was analyzed using the Wilcoxon matched-pairs signed-rank test. **(D)** Kaplan-Meier analysis of the overall survival of 92 NSCLC patients (data from TMA cohort 3). **(E)** IHC results revealed that the expression of QSOX2 was positively correlated with TNM stage and lymph node metastasis status in NSCLC. **(F)** Violin plot comparing the expression of *QSOX2* between NSCLC and non-tumor tissue samples from the TCGA dataset. **(G)** Kaplan-Meier survival curves plotted by R Studio (survival package) according to the different expression levels (normal, slightly elevated, moderately elevated, and significantly elevated) of *QSOX2* in stage III–IV NSCLC samples.

Previous studies have demonstrated that *QSOX2* plays a key role in regulating the sensitization of neuroblastoma cells to IFN-gamma induced apoptosis (Wittke et al., 2003). However, to the best of our knowledge, the expression level and function of the *QSOX2* gene in NSCLC and other malignant tumors have not been investigated. To elucidate the expression of the *QSOX2* gene in NSCLC and its impacts on survival and clinicopathological features, we extracted mRNA from the resected tumor tissue samples from 22 patients in cohort 1 with NSCLC and then analyzed by qPCR. The results showed the mRNA level of *QSOX2* was significantly elevated in the tumor tissue samples (*P* = 0.00083) (Figure 1A). Next, we examined the protein expression of QSOX2 in paraffin-embedded NSCLC samples from 34 other patients using IHC. Representative images of the IHC results are shown in Figure 1B. Significant differences in QSOX2 protein expression were found between the NSCLC and adjacent tissue samples, and overexpression of QSOX2 was observed in the tumor tissue samples (Figures 1B,C). TMA from an independent NSCLC cohort was used to determine whether the expression of *QSOX2* is correlated with clinical prognosis in NSCLC. The patients were divided into two groups according to the expression level of *QSOX2*. Kaplan-Meier analysis with the log-rank test revealed that NSCLC patients with higher levels of this protein had significantly lower survival rates than patients with lower protein levels (Figure 1D). Elevated expression of *QSOX2* was correlated with lymph node metastasis and an advanced tumor-node-metastasis (TNM) stage in NSCLC (Figure 1E and Table 1). Lastly, we validated these findings in publicly available TCGA-NSCLC data, *QSOX2* was upregulated in NSCLC tissues (*n* = 1019) compared to adjacent normal tissues (*n* = 110) (*P* = 8.003 × 10^–62^). Moreover, overexpression of *QSOX2* in stage III-IV patients was associated with a poor prognosis (*P* = 0.043) (Figures 1F,G). Furthermore, as shown in Supplementary Figures 1A,B, we found that *QSOX2* mRNA expression was higher in the 35 primary tumor types compared with the normal group in Pan-TCGA database (*P* = 3.474 × 10^–175^). Survival analysis showed that as the expression of *QSOX2* increased, the prognosis worsened. Taken together, these results illustrate that high level of *QSOX2* is expressed in NSCLC and indicative of a poor prognosis.

**TABLE 1:** Association of *QSOX2* expression levels with clinicopathologic characteristics in NSCLC.

		QSOX2 Expressed level				
		**Low**		**High**		
		**Count**	**N%**	**Count**	**N%**	***P*-value**

Sex	Male	26	28.26%	25	27.17%	
	Female	24	26.09%	17	18.48%	0.470
Age	≤60 years	21	22.83%	17	18.48%	
	>60 years	29	31.52%	25	27.17%	0.882
Pathological grade	(I, I–II, II)	34	37.36%	26	28.57%	
	(II–III, III)	15	16.48%	16	17.58%	0.453
T	T1–T2	36	45.57%	24	30.38%	
	T3–T4	9	11.39%	10	12.66%	0.332
N	Negative	23	33.33%	11	15.94%	
	Positive	14	20.29%	21	30.43%	**0.021**
AJCC Stage	I–II	29	43.28%	12	17.91%	
	III–IV	10	14.93%	16	23.88%	**0.009**
ALK (FISH)	Negative	43	51.19%	31	36.90%	
	Positive	4	4.76%	6	7.14%	0.279
EGFR (FISH)	Negative	34	36.96%	37	40.22%	
	Positive	16	17.39%	5	5.43%	**0.022**

*Bold values indicate less than 0.05.*

### Secretion of *QSOX2* Is Upregulated in NSCLC and Sensitive to Tumor Suppression

**FIGURE 2:**
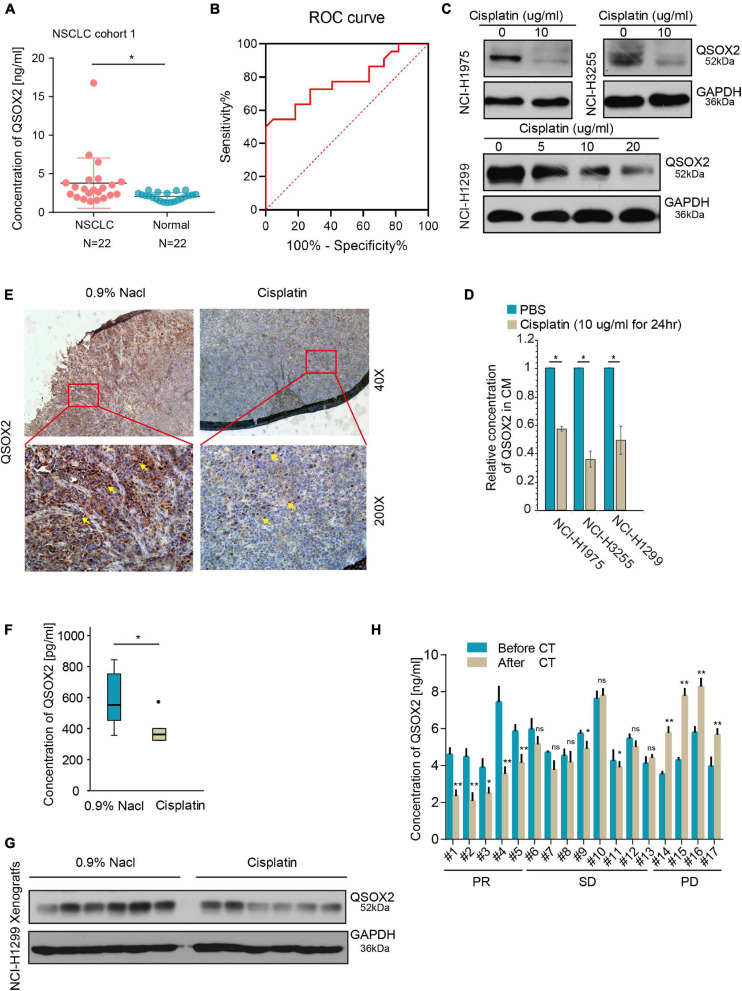
Quiescin sulfhydryl oxidase 2 (*QSOX2*) can be used as a circulating marker to monitor tumor growth. **(A)** Serum samples were collected from 22 patients in cohort 1 and 22 healthy persons and subjected to ELISA analysis for QSOX2 expression; *P < 0.05. **(B)** ROC curve analyses of QSOX2 signature to discriminate NSCLC patients from healthy persons. **(C)** NCI-H3255 and NCI-H1975 cells were treated with cisplatin (10 μg/ml) for 24 h, NCI-H1299 cells were treated with a graded dose of cisplatin (5/10/20 μg/ml) for 24 h, and all cells were harvested for WB analysis with an anti-QSOX2 antibody. **(D)** Concentrated cell culture medium supernatant from panel **(C)** was harvested and subjected to ELISA analysis for QSOX2 expression; *P < 0.05. **(E)** Representative *QSOX2*-stained sections from 0.9% NaCl- or cisplatin (5 mg/kg)-treated xenografts are shown. The yellow arrow represents QSOX2. **(F)** The boxplot compares the QSOX2 level in the serum of tumor-bearing mice treated with or without cisplatin; *P < 0.05. **(G)** The xenografts from were harvested for Western blot (WB) analysis with the anti-QSOX2 antibody. **(H)** The expression levels of QSOX2 were compared before and after 2 cycles of cisplatin-based chemotherapy. Then, sera were harvested from 17 NSCLC patient and subjected to ELISA and WB analysis for QSOX2 expression, *P < 0.05 and **P < 0.01.

Previous studies have shown that *QSOX2* can be expressed intracellularly and secreted extracellularly (Kuo et al., 2017). Thus, we postulated that the level of QSOX2 is elevated in the serum of NSCLC patients. To test this hypothesis, serum samples from 22 healthy people and 22 patients with stage III-IV NSCLC (cohort 1) were collected, and the protein concentrations of QSOX2 in these serum samples were determined by ELISA. As shown in Figure 2A, the concentration of QSOX2 in the serum samples of 12 out of 22 of the patients was higher than the average concentration in the normal control serum samples, suggesting increased secretion of QSOX2 by tumors into the serum. Based on depicted ROC (AUC = 0.7769, specificity = 0.636, sensitivity = 0.818), QSOX2 exerted diagnostic potential for NSCLC (Figure 2B). To determine whether the expression of QSOX2 in tumor and circulating body fluid was down-regulated after cisplatin treatment, we performed the following *in vitro* and *in vivo* experiments. First, we detected changes in QSOX2 expression in NSCLC cells and their culture medium after 24 h of cisplatin treatment using WB and ELISA methods, respectively. The results showed that the intracellular and extracellular concentrations of the QSOX2 protein in supernatant of NCI-H1975, NCI-H3255 or HCI-H1299 cell cultures after cisplatin treatment were significantly reduced (Figures 2C,D). Next, in cisplatin treated NCI-H1299-xenograft mouse model (Supplementary Figures 2A,B), IHC, WB, and ELISA analyses indicated that the concentrations of QSOX2 in tumor cells and host serum samples were significantly decreased after cisplatin treatment (Figures 2E–G). Clinically, we collected serum samples from 17 patients and found that QSOX2 was highly expressed in serum from those with stage IIIB–IVC NSCLC before and after 2 cycles of cisplatin-based treatment, as shown by ELISAs. According to the RECIST criteria, partial response (PR), stable disease (SD), and progressive disease (PD) were noted in 5, 9, and 4 cases, respectively. The results showed that QSOX2 was significantly decreased after cisplatin-based treatment in PR cases, slightly fluctuated in SD patients, and significantly increased in PD patients (Figure 2H). Six samples with different responses to chemotherapy were randomly selected and verified by WB (Supplementary Figure 2C), and the results were consistent with the above findings. These results suggest that the expression of exocrine QSOX2 protein increased in the serum of NSCLC patients, and downregulation of intracellular and extracellular QSOX2 expression was related to the anti-tumor therapy.

### *QSOX2* Contributes to the Proliferation, Cell Cycle Progression and Survival of NSCLC Cells

**FIGURE 3:**
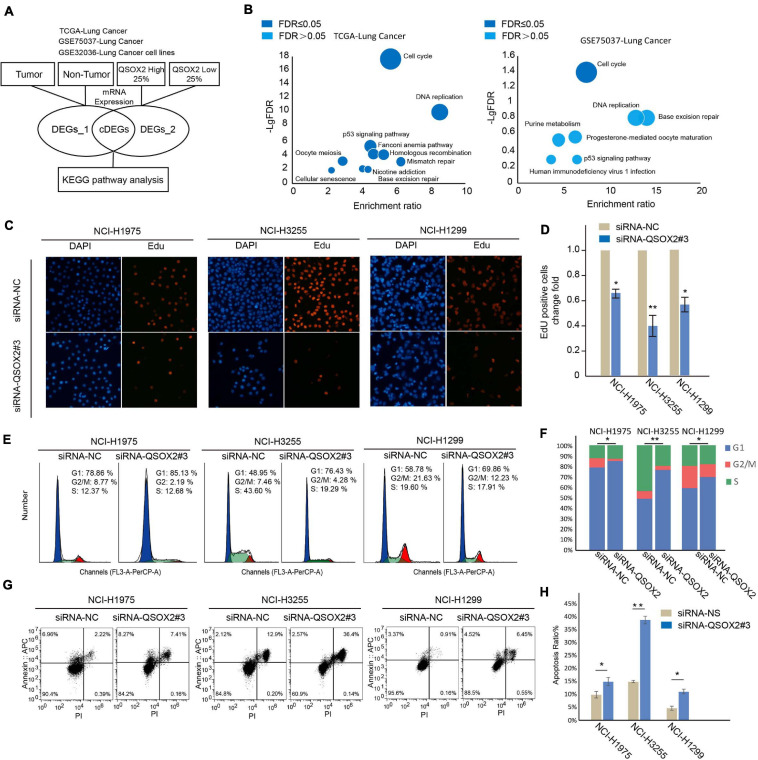
Downregulation of *QSOX2* expression represses the proliferation, cell cycle and survival of NSCLC cells. **(A)** DEG and KEGG pathway analyses of TCGA and GEO datasets. The bioinformatic data analysis workflow consisted of three publicly available datasets. **(B)** KEGG enrichment bubble chart. Negative log base 10 FDR values from the pathway enrichment analysis are plotted. Dark blue: FDR < 0.05; light blue: FDR ≥ 0.05. **(C,D)** NSCLC cells transfected with the indicated plasmids and subjected to EdU assays. Quantitative analysis was performed using ImageJ software. The bar graph shows the mean ± SD; *n* = 3, *P < 0.05, **P < 0.01. **(E,F)** NSCLC cells transfected with the indicated plasmids for 48 h and subjected to cell cycle assays. The percentage of cells in each phase of the cell cycle was quantified in an accumulated histogram. *P < 0.05. **(G,H)** NSCLC cells transfected with the indicated plasmids and subjected to apoptosis assays. Quantitative analysis was performed using FlowJo software. The bar graph shows the mean ± SD; *n* = 3, *P < 0.05, **P < 0.01.

To explore the molecular mechanism leading to a poor prognosis in tumors with high expression of the *QSOX2* gene, we conducted a bioinformatic analysis (Figure 3A) to mine the relevant signaling pathways. Two databases containing transcriptional profiles of lung cancer and adjacent tissue samples (TCGA-Lung Cancer and GSE75037-Lung Cancer) and one database containing expression profiles of lung cancer cell lines and non-cancer cell lines (GSE32036-Lung Cancer) were selected for further analysis. The differentially expressed genes (DEGs) derived from comparing the cancer and non-cancer cells and the high and low *QSOX2* expression tumors were intersected, and the shared genes were further analyzed for Kyoto Encyclopedia of Genes and Genomes (KEGG) pathway enrichment analysis. As shown in Figure 3B and Supplementary Figure 3A, the results suggested that all the *QSOX2*-related genes from the three independent databases were enriched in the cell cycle pathway (false discovery rate (FDR) ≤ 0.05). To validate the conclusions of the above bioinformatic analysis, we conducted a set of cell proliferation, cell cycle and apoptosis assays using engineered siRNAs that reduced the expression of *QSOX2* in NCI-H1299, HCI-H3255, and NCI-H1975 cells. *QSOX2* knockdown dramatically reduced the growth rates of the NCI-H1975, NCI-H3255, and NCI-H1299 cells (Figures 3C,D and Supplementary Figures 3B–D). Moreover, knocking down *QSOX2* expression resulted in the accumulation of cells in G1 and decreased frequencies of cells in the S and G2/M phases (Figures 3E,F). Furthermore, *QSOX2* knockdown resulted in a significant increase in the percentage of apoptotic cells (Figures 3G,H). These results indicate that *QSOX2* is important for cell cycle progression in NSCLC cells and that repression of *QSOX2* accelerates cell apoptosis.

### *QSOX2* Is a Periodic Gene That Regulates the Cell Cycle and Wnt Pathway

**FIGURE 4:**
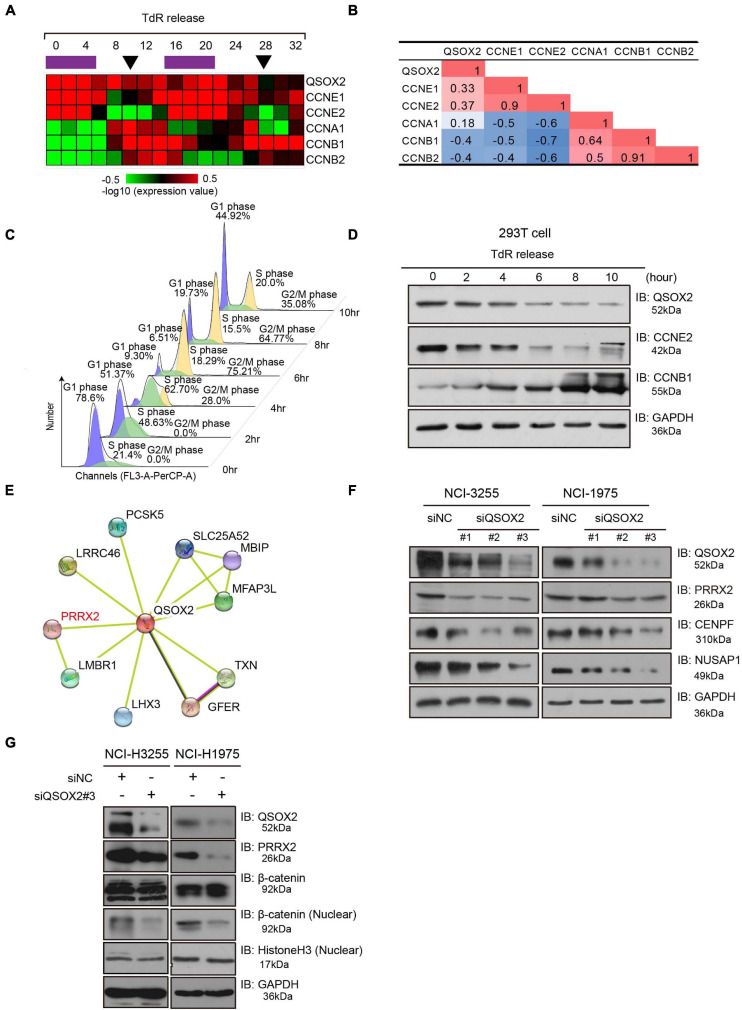
Quiescin sulfhydryl oxidase 2 (*QSOX2*) is periodically expressed during the cell cycle. **(A)** Bioinformatic data analysis of GSE52100. Expression heatmap of *QSOX2* and cyclin genes in two cell cycle courses after double thymidine block release. The purple bars indicate S phase, and the black arrows indicate mitosis. **(B)** Correlation analysis between *QSOX2* and cyclin gene expression; red: positive correlation; blue: negative correlation. **(C)** 293T cells synchronized with a double thymidine block, released and evaluated at several time points. The cell cycle profiles were assessed by flow cytometry after propidium iodide (PI) staining of DNA. **(D)** The expression of *QSOX2* and cyclin genes in the cell lysates from panel **(C)** assessed by Western blot analysis. **(E)** Protein–protein interaction network (PPI) analysis of QSOX2 assessed by STRING (http://string-db.org/) **(F)** WB analysis of NCl-H3255 and NCl-H1975 cells transfected with siRNA-control or siRNA-*QSOX2* (48 h) and evaluated with the indicated antibodies. **(G)** Immunoblotting of β-catenin in the nuclear extracts of NCl-H3255 and NCl-H1975 cells was carried out after *QSOX2* silencing, and GAPDH and Histone 3 were used as a loading controls, respectively.

**FIGURE 5:**
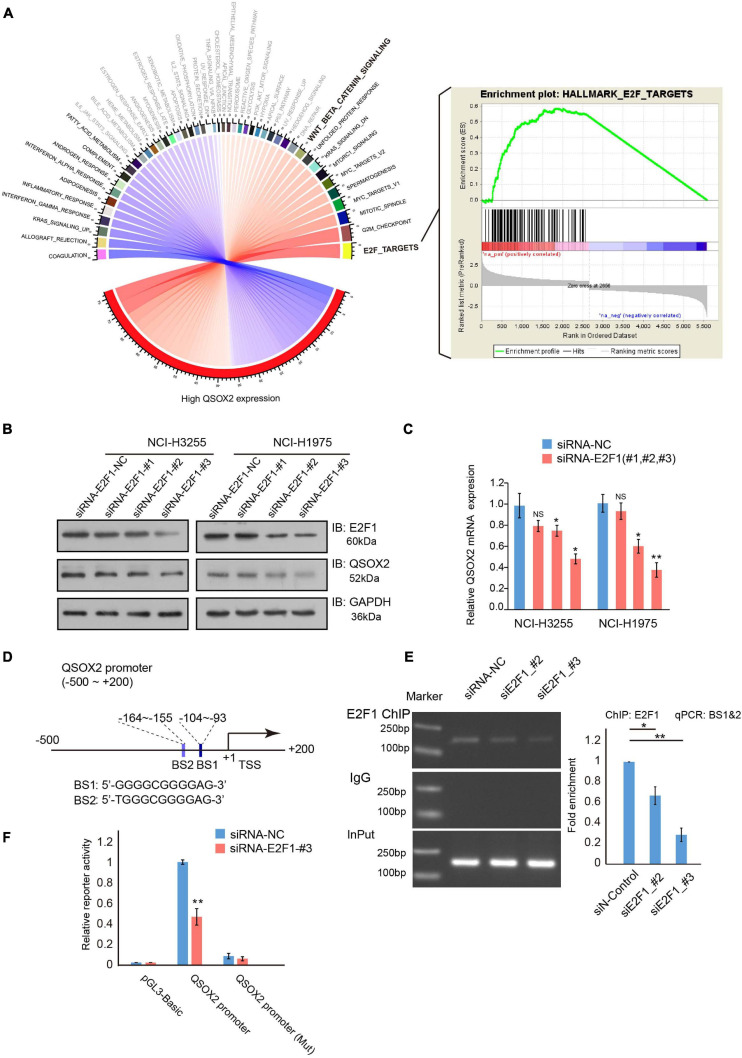
E2F transcription factor 1 (*E2F1*) regulates *QSOX2* expression directly. **(A)** Chord diagram of all enriched hallmark gene sets in a GSEA. Black font: FDR < 0.05; Gray font: FDR ≥ 0.05; Blue curve: negative enrichment; Red curve: positive enrichment. **(B)** WB analysis of NCI-H3255 and NCI-H1975 cells transfected with shRNA-control or shRNA-E2F1 (48 h) and evaluated with the indicated antibodies. **(C)** NCI-H3255 and NCI-H1975 cells transfected with the indicated plasmids and subjected to real-time PCR analysis for *E2F1* and *QSOX2* expression. *P < 0.05, **P < 0.01. **(D)** Schematic diagram of the putative bonding sites for E2F1 in the QSOX2 promoter region. BS1: binding site 1; BS2: binding site 2. **(E)** Chip-qPCR analysis of E2F1 binding to the promotor of QSOX2 in 293T cells after treatment with siRNA-NC or siRNAs-E2F1. Purified rabbit IgG was used as a negative control for the background enrichment signal; *P < 0.01; **P < 0.001. **(F)** Co-transfection of the QSOX2 promoter and siRNAs-E2F1 into 293T cells in triplicate. Relative *QSOX2* promoter activities were measured at 48 h after transfection by a dual-luciferase assay; *P < 0.05, **P < 0.01.

Quiescin sulfhydryl oxidase 2 is related and contributes to the cell cycle process. However, it is still unclear whether *QSOX2* is expressed periodically in different phases of the cell cycle, and the function of *QSOX2* in cell cycle regulation needs to be further studied. To determine whether *QSOX2* is a cell cycle-related protein, a dataset from the GEO database (GSE52100) containing cell cycle-regulated mRNA transcripts from the entire genome of the osteosarcoma-derived U2OS cell line was downloaded, and we compared the expression levels of *QSOX2* and classic periodic marker genes (*CCNE1*, *CCNE2*, *CCNA1*, *CCNB1*, and *CCNB2*) in different phases of cell cycle progression after double thymidine block release. Interestingly, the expression of *QSOX2* varied periodically in the different phases of the cell cycle, with increased expression observed in the G1/S phase and decreased expression found in the G2/M phase (Figure 4A). Correlation analysis showed that the expression of *QSOX2* was positively related to the expression of *CCNE1* and *CCNE2* but negatively correlated with that of *CCNB1* and *CCNB2* (Figure 4B). These results were verified in 293T cells by *in vitro* experiments. As shown in Figures 4C,D, we arrested 293T cells at the G1/S transition using a double thymidine block, followed by a release into fresh medium to allow the cells to progress through the cell cycle. The degree of synchrony in each cell cycle phase was monitored by flow cytometry analysis after the thymidine block. *QSOX2* expression peaked in the G1 phase, and before mitosis, QSOX2 protein expression dropped sharply and remained at low levels until the late in the following G1 phase. To preliminarily explore the molecular mechanism by which *QSOX2* participates in and regulates the cell cycle of NSCLC cells, we conducted a protein–protein interaction (PPI) analysis of QSOX2 using STRING^6^. As a result, a total of 10 genes potentially interacting with QSOX2 were included in the PPI network (Figure 4E). Among these candidates, PRRX2 captured our attention because it is overexpressed in highly invasive and metastatic malignant tumors. Downregulation of PRRX2 expression prevented the nuclear translocation of β-catenin and inhibited the Wnt/β-catenin signaling pathway (Chai et al., 2019). We observed that repression of QSOX2 could decrease the expression of CENPF, NUSAP1, PRRX2, and β-catenin distribution in the nucleus, with the former two proteins being cell division-associated proteins (Figures 4F,G), and gene set enrichment analysis (GSEA) confirmed that Wnt and β-catenin signaling genes were significantly enriched in the tumors with high QSOX2 expression compared to those with low QSOX2 expression (Figure 5A). Taken together, these results demonstrate that *QSOX2* is expressed periodically and exhibits increased expression in the G1 phase of the cell cycle; silencing *QSOX2* could repress the expression of cell division-associated proteins and block PRRX2-Wnt signing.

### E2F1 Directly Regulates QSOX2 by Specifically Recognizing the QSOX2 Promoter

Next, we determined the underlying mechanism of the regulation of *QSOX2* expression. GSEA of hallmark gene sets was conducted to identify phenotypic signature in the high *QSOX2* expression group of the TCGA-NSCLC cohort. The results showed that in the high *QSOX2* group, all the *E2F* target genes were enriched (Figure 5A), indicating that the expression of *QSOX2* may be regulated by E2F family members. Previous studies have confirmed that E2Fs function as major transcriptional regulators of cell cycle-dependent gene expression, and the levels of the activator protein E2F1 peak during the G1/S phase transition (Kent and Leone, 2019). To determine whether E2F1 can promote the transcription of QSOX2, we conducted an *E2F1* knockdown experiment. The results showed that siRNA-E2F1 decreased both the mRNA and protein levels of QSOX2 in NCI-H3255 and NCI-H1975 cells (Figures 5B,C). Thus, we suspected that E2F1 regulated QSOX2 transcription through a direct mechanism. Supporting this idea, two potential E2F1-binding sites (BS1: −164 ∼−155 and BS2: −104 ∼−93) were identified in the QSOX2 promoter (Figure 5D). To test this idea, we conducted a ChIP-PCR assay in 293T cells and found that E2F1 bound to the BS1∼2 region of the QSOX2 promoter, whereas silencing E2F1 decreased the abundance of E2F1 at the QSOX2 promoter (Figure 5E). To confirm the ChIP results, we constructed luciferase reporter vectors driven by the E2F1 binding site contained in the QSOX2 promoter and performed luciferase reporter assays using 293T cells. As shown in Figure 5F, E2F1 knockdown markedly decreased the luciferase activity driven by the BS1∼2-containing QSOX2 promoter but not that driven by a mutant BS1∼2-containing QSOX2 promoter in 293T cells. These results demonstrate that E2F1 directly promotes the transcription of QSOX2 during the cell cycle.

### E2F1 Expression Is Positively Associated With *QSOX2* in the NSCLC Clinical Cohort

**FIGURE 6:**
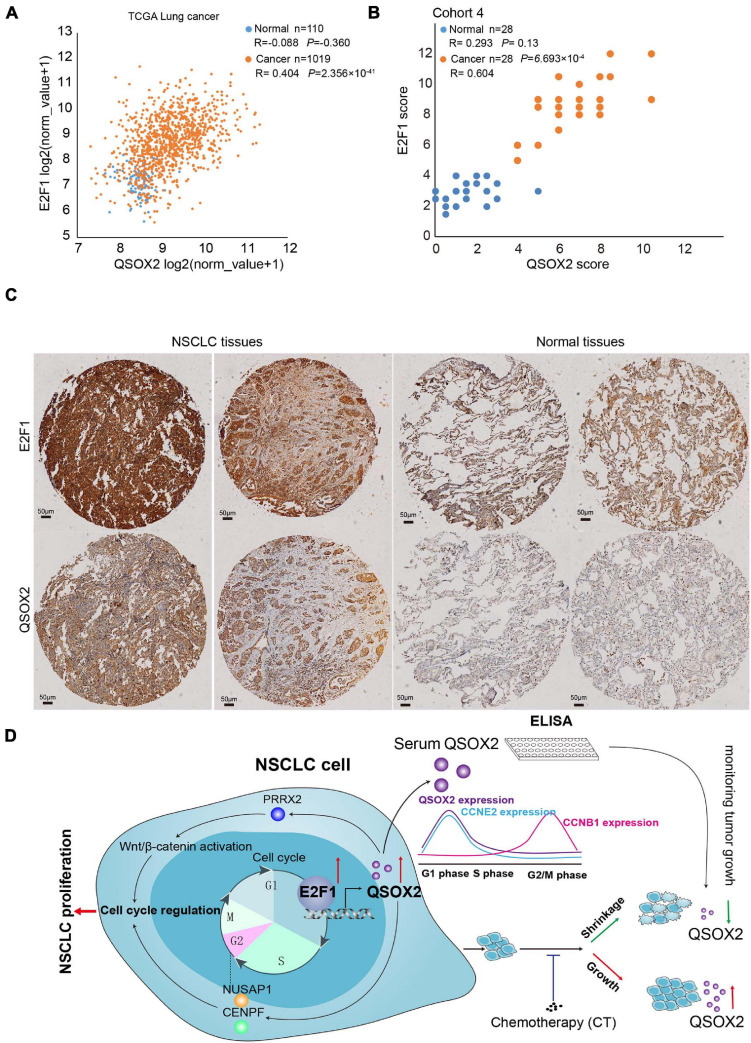
Quiescin sulfhydryl oxidase 2 (*QSOX2*) expression is positively correlated with E2F1 expression in NSCLC tissue. **(A)** Scatter plot for analyzing the correlation between E2F1 expression and *QSOX2* expression in NSCLC (orange dot) and non-tumor (blue dot) tissue samples from a TCGA dataset. **(B)** Scatter plot for analyzing the correlation between E2F1 expression and *QSOX2* expression in NSCLC (orange dot) and non-tumor (blue dot) tissue samples from the 28 NSCLC patients (data from TMA cohort 4). **(C)** IHC detection of QSOX2 and E2F1 expression in representative NSCLC samples and Normal tissues. **(D)** A schematic model showing our findings in this study.

To validate the result found in aforementioned *in vitro* experiments in the clinical cohort, we analyzed the mRNA levels of *E2F1* and *QSOX2* in TCGA-NSCLC datasets using bioinformatic analysis and examined the protein levels of these molecules in primary NSCLC samples and matched adjacent normal lung tissue samples (28 pairs in NSCLC cancers cohort 4) by IHC. As a negative control, there was no positive expression of proteins in the tumor tissues (Supplementary Figure 4). In agreement with the previous results, *E2F1* and *QSOX2* were highly expressed in the NSCLC tissue samples but not in the paired adjacent normal tissue samples (Figures 6A,B). Interestingly, E2F1 expression was positively correlated with the expression of QSOX2 in the tumor tissue samples. However, in the normal tissue samples, there was no significant correlation between the expression of E2F1 and QSOX2 (Figures 6B,C).

## Discussion

Inhibition of tumor proliferation is an effective response to cytotoxic drug therapy. However, inflammatory necrosis-induced pseudoincreases in tumor volume and non-expression of specific serum tumor markers in some cancer patients have biased the evaluation of therapeutic efficacy after anticancer therapies (Ohashi et al., 2007; Ichihara et al., 2008). Identification of a serum-specific tumor marker that can dynamically and truly reflect the degree of cell cycle inhibition in tumor cells is urgently needed. Such a serum marker may be relatively helpful in evaluating the efficacy of chemotherapy. In this study, we showed that QSOX2, a secreted protein, was aberrantly expressed in NSCLC and periodically expressed in the cell cycle process of tumor cells, which implied that this protein might be a promising serum marker for the evaluation of tumor suppression after anticancer therapy.

The QSOX protein is a flavoprotein responsible for converting sulfhydryl-containing substances into the corresponding disulfide-containing substance at the expense of molecular oxygen and generation of hydrogen peroxidase (Raje and Thorpe, 2003). Two genes, *QSOX1* and *QSOX2*, encode the QSOX proteins. It has been shown that QSOX1 is a key enzyme in the ability of tumors to modify the ECM, which enables tumors to communicate with and/or modify their environment at the tumor-stroma interface, contributing to the invasion and subsequent growth of the tumor (Ilani et al., 2013). Previous studies have shown that QSOX1 is overexpressed in tumor tissues and promotes the invasion and metastasis of HCC (Zhang et al., 2019), lung cancer (Sung et al., 2018), prostate cancer (Baek et al., 2018), and breast cancer cells (Hollern et al., 2014). Additional evidence has shown that breast cancer with high expression of QSOX1 has a poor prognosis but that inhibition of QSOX1 expression can effectively inhibit the invasion and distant metastasis of pancreatic and renal cancer cells (Hanavan et al., 2015). For *QSOX2*, only one study has assessed the relationship between *QSOX2* and cancer. In neuroblastoma, *QSOX2* is a major player in regulating the sensitivity of neuroblastoma cells to IFN-γ-induced apoptosis (Wittke et al., 2003). In this study, we showed that *QSOX2* expression was upregulated in NSCLC tissues, and high expression of *QSOX2* was associated with a poor tumor prognosis, lymph node metastasis and an advanced TNM stage. In addition, bioinformatics analysis based on TCGA data supported our research findings.

A recent study demonstrated the presence of the secreted (short form) and transmembrane (long form) forms of QSOX2, which exhibited distinct molecular weights, with the long form exhibiting a protein molecular weight at ∼75 kDa and the short form having a molecular weight of ∼50 kDa (Kuo et al., 2017). The same results were found by our WB assay, and the expression of the short form in NSCLC cell lines was significantly higher than that of the long form. This finding suggests that secreted *QSOX2* may be detected in NSCLC cell culture medium or tumor patient serum. Consistent with our hypothesis, in selected NSCLC patients, 60% of the patients had higher QSOX2 serum levels than the normal population. We are interested in whether such a serum tumor marker can be used to monitor the tumor suppression after anticancer therapy. The results of *in vitro* and *in vivo* studies showed that after cisplatin treatment, the expression of exocrine and intracellular *QSOX2* by NSCLC cells decreased significantly as the tumor cells underwent apoptosis and the xenograft shrunk. Our results suggest a possible role for *QSOX2* as a circulating marker to monitor tumor suppression in NSCLC patients. A previous study verified the serum level of miR-125b as a dynamic monitoring marker for evaluating the chemotherapy response in NSCLC patients (Cui et al., 2013).

To uncover the molecular mechanism underlying abnormal activation of *QSOX2*, we analyzed three NSCLC expression profile databases from GEO, one of which contained NSCLC cell line expression data used to avoid the expression profiles of stromal cell components biasing the analysis results. Differential gene expression and KEGG pathway analyses showed that QSOX2 mainly acted on the cell cycle pathway. Deregulation of the cell cycle is one of the most frequent alterations during tumor development, and the sustained proliferation caused by this deregulation is one of the hallmarks of cancer (Hanahan and Weinberg, 2011). Our vitro experiments found that knockdown of *QSOX2* expression by siRNA inhibited cell proliferation and significantly increased apoptosis. Interestingly, *QSOX2* silencing resulted in a significant reduction in the proportion of NSCLC cells in the S and G2/M phases of the cell cycle, and the expression of mitosis-associated proteins (CENPF and NUSAP1) was also significantly decreased in the *QSOX2*-inhibited group. These results suggest that the expression of *QSOX2* is essential for the abnormal regulation of the tumor cell cycle and tumor cell survival. Cell cycle synchronization assays are widely used to study periodically expressed proteins and their functions during cell cycle progression (Grant et al., 2013; An et al., 2019). In this study, after release from double thymidine block, Western blotting showed that the variation in *QSOX2* expression was consistent with that in *CCNE2* expression but not that in *CCNB1* expression, indicating that *QSOX2* is regulated during the cell cycle and functions in the G1 phase. Further experiments showed that *QSOX2* could regulate the expression of PRRX2 and nuc-β-catenin, which play important roles in the activation of the Wnt signaling pathway (Chai et al., 2019). This finding was further supported by WB and GSEA, which also indicated the ability of *QSOX2* to indirectly regulate Wnt pathway activity in tumor cells and thus affect tumor proliferation.

The E2F family plays an important role in forming the core transcriptional machinery driving cell cycle progression. For E2F proteins, the levels of activation proteins (E2F1 and E2F2) peak during the G1 phase, those of atypical repressors (E2F7 and E2F8) peak late in the S phase, and those of typical repressors (E2F3B, E2F4, E2F5, and E2F6) remain consistent throughout the cell cycle (Kent and Leone, 2019). *E2F1* was the first cloned gene in the E2F family and initiates the transcription of target genes by recognizing and binding to conserved sequences in the promoter regions of downstream genes (Kwon et al., 2017). In our study, GSEA found that E2F target genes enriched in the high *QSOX2* expression group had the most significant FDR values, and similar to that of E2F1, the expression of *QSOX2* peaked in the G1 phase. According to cell line experiments, the expression level of *QSOX2* decreased significantly after silencing E2F1. Furthermore, we confirmed that the promotor of QSOX2 was occupied by E2F1 using ChIP analysis, and when E2F1 was silenced, luciferase reporter gene analysis revealed that the E2F1 binding signal in the QSOX2 promoter region was suppressed. Our results demonstrate that E2F1 directly regulates QSOX2 transcription. Interestingly, we found that *E2F1* and *QSOX2* exhibited highly elevated expression and were positively correlated with each other in primary human NSCLC samples. However, the expression of *E2F1* and *QSOX2* was not correlated in the corresponding adjacent tissue samples. This difference can be explained by a previous study, which concluded that E2F activators might be dispensable for normal cell proliferation in healthy adult tissues (Kent and Leone, 2019). Hence, in agreement with other studies, our study suggests that *QSOX2* expression is essential for E2F1 to regulate cell cycle progression. Moreover, the expression level of *QSOX2* reflects the activity of the tumor cell cycle and more specifically the transcriptional activity of E2F1 more specifically.

## Conclusion

In summary, our results indicated that *QSOX2* was overexpressed in NSCLC and could serve as a potential prognostic biomarker (Figure 6D). Our results also suggested that *QSOX2* could serve as a circulating marker for evaluation of tumor suppression after anti-cancer therapy. In addition, we demonstrated for the first time that *QSOX2* acted as an essential periodically expressed gene by regulating the cell cycle in NSCLC, and its silencing significantly inhibited the proliferation of NSCLC cells. Mechanistically, *QSOX2* expression was directly upregulated by the transcription factor E2F1. A future large clinical study is warranted to determine the utility of *QSOX2* as an non-invasive biomarker for monitoring tumor burden.

## Data Availability Statement

The datasets presented in this study can be found in online repositories. The names of the repository/repositories and accession number(s) can be found in the article/Supplementary Material.

## Ethics Statement

This work was approved by the Ethics Committee of the First Affiliated Hospital of Nanchang University (no. MR2018-83). Written informed consent for participation was not required for this study in accordance with the national legislation and the institutional requirements. The animal study was reviewed and approved by the First Affiliated Hospital of Nanchang University (no. MR2018-83).

## Author Contributions

CS: conception and design. YL, ML, ZXZ, and CS: acquisition of data. HL and YW: clinical samples and information collection. YL, ML, CS, and YW: conduction of experiments. LD, ZYZ, and CZ: analysis and interpretation of data. YL and CS: writing, and revision of the manuscript. CS and JX: study supervision. All authors read and approved the manuscript.

## Conflict of Interest

The authors declare that the research was conducted in the absence of any commercial or financial relationships that could be construed as a potential conflict of interest.

## Footnotes

1 www.superchip.com.cn/biology/tissue.html

2 http://xena.ucsc.edu/

3 http://www.r-project.org

4 www.webgestalt.org

5 http://jaspar.genereg.net/

6 www.string-db.org

## Supplementary Material

Supplementary Figure 1 Overexpression of *QSOX2* in malignancies correlates with prognosis. **(A)** The expression of *QSOX2* in non-tumor (*n* = 707) and tumor (*n* = 10,323) tissue samples, according to the Pan-TCGA dataset. **(B)** The association between the expression level of *QSOX2* and patient survival, according to overall survival time data in the Pan-TCGA dataset.

Supplementary Figure 2 The cisplatin treated NCI-H1299-xenograft mouse model and clinical samples. **(A)** The graph shows xenograft growth with or without cisplatin treatment. **(B)** The boxplot compares the volumes of xenografts treated with or without cisplatin. **(C)** The sera from 6 patients were diluted 15 times and subjected to WB analysis for QSOX2 expression. Reversible Ponceau S staining was applied to ensure equal loading of the gels. **P* < 0.05.

Supplementary Figure 3 Kyoto Encyclopedia of Genes and Genomes pathway enrichment analysis of *QSOX2*-related genes. **(A)** KEGG enrichment bubble chart. Negative log base 10 FDR values from the pathway enrichment analysis were plotted. Dark blue: FDR < 0.05; light blue: FDR ≥ 0.05. **(B–D)** NSCLC cells transfected with the indicated plasmids, and the cell numbers were measured at the indicated time points using flow cytometry. The data shown represent the mean ± SD, *n* = 3, ^∗∗^*P* < 0.01.

## Abbreviations

BS: binding site
TSS: transcription start sites
CCK-8: cell counting kit-8
CCNB1: cyclin B1
CENPF: centromere protein F
QSOX2: quiescin sulfhydryl oxidase 2
E2F1: E2F transcription factor 1
CCNE2: cyclin E2
NUSAP1: nucleolar and spindle associated protein 1
PRRX2: paired mesoderm homeobox protein 2
ChIP: chromatin immunoprecipitation
EdU: 5-ethynyl-2-deoxyuridine
GAPDH: glyceraldehyde 3-phosphate dehydrogenase
GEO: gene expression omnibus
NSCLC: non-small cell lung cancer
PBS: phosphate-buffered saline
siRNA: small interfering RNA
TCGA: The Cancer Genome Atlas
TNM: tumor-node-metastasis.
