# Supplementary figures and images for: QSOX2 Is an E2F1 Target Gene and a Novel Serum Biomarker for Monitoring Tumor Growth and Predicting Survival in Advanced NSCLC
Source: Front Cell Dev Biol. 2021 Jul 19;9:688798. doi: 10.3389/fcell.2021.688798 (PMC8326667; doi:10.3389/fcell.2021.688798)

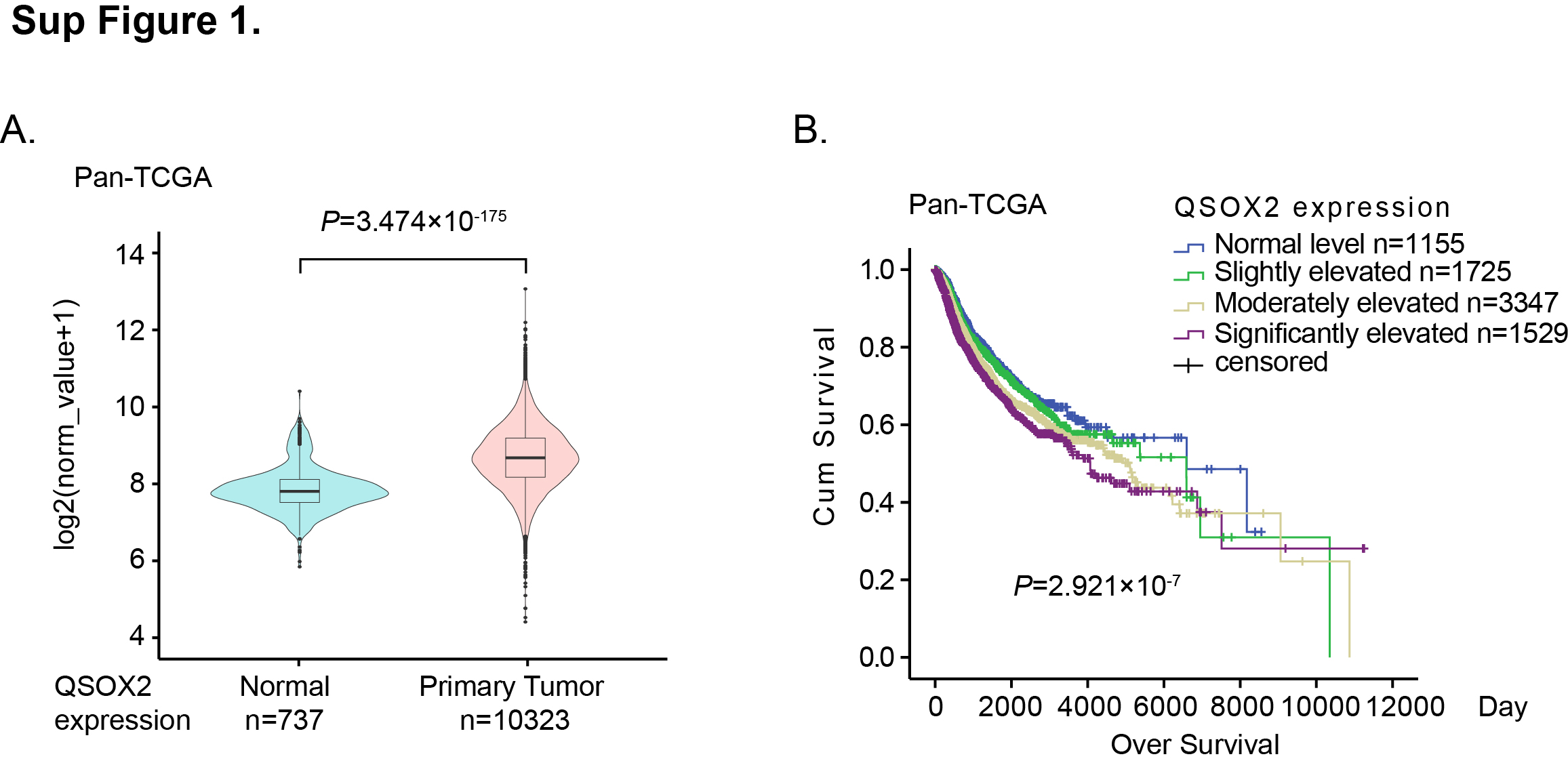

Supplement: Supplementary Figure 1 — Overexpression of QSOX2 in malignancies correlates with prognosis. (A) The expression of QSOX2 in non-tumor (n = 707) and tumor (n = 10,323) tissue samples, according to the Pan-TCGA dataset. (B) The association between the expression level of QSOX2 and patient survival, according to overall survival time data in the Pan-TCGA dataset. [file Image_1.JPEG]

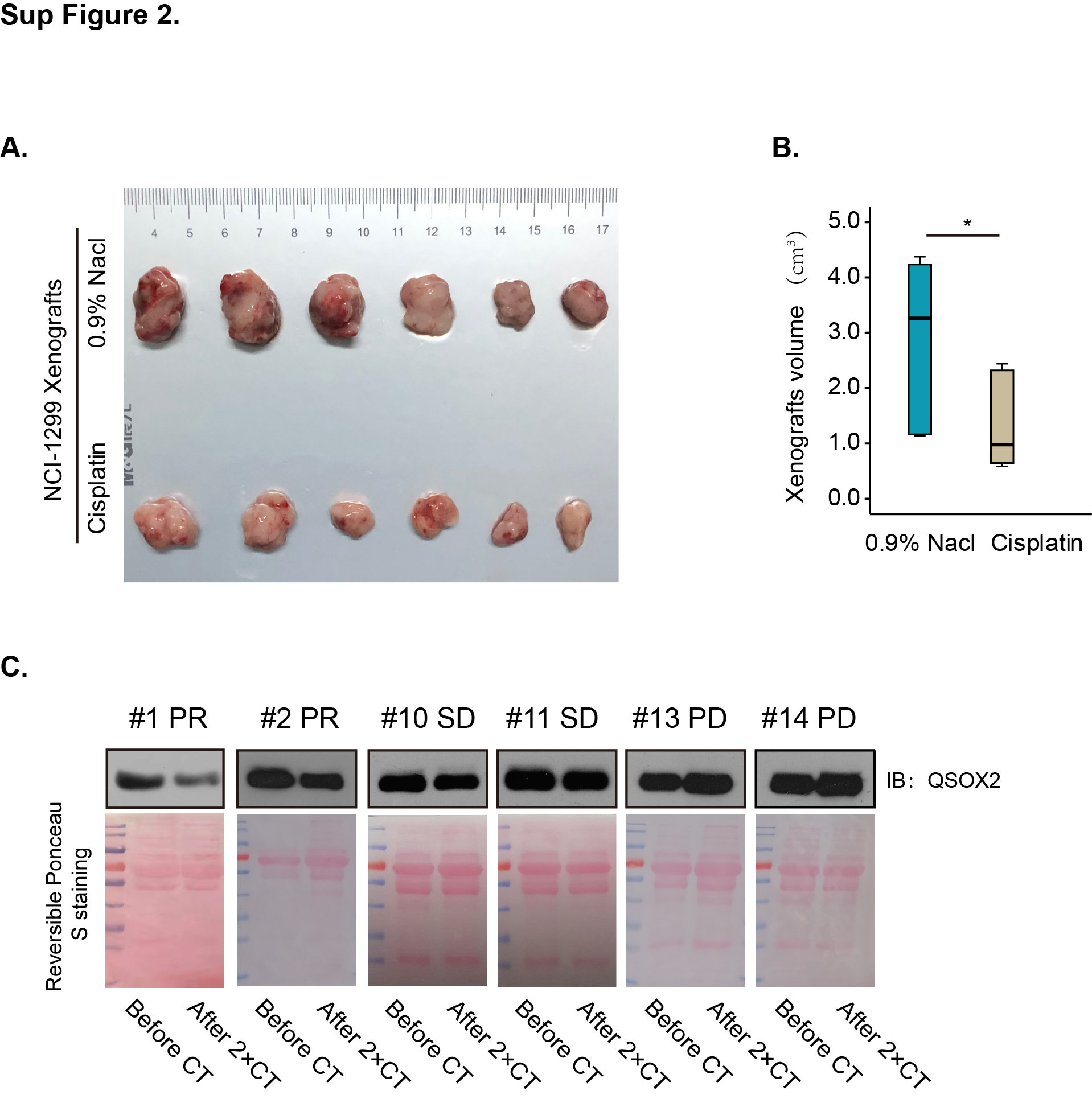

Supplement: Supplementary Figure 2 — The cisplatin treated NCI-H1299-xenograft mouse model and clinical samples. (A) The graph shows xenograft growth with or without cisplatin treatment. (B) The boxplot compares the volumes of xenografts treated with or without cisplatin. (C) The sera from 6 patients were diluted 15 times and subjected to WB analysis for QSOX2 expression. Reversible Ponceau S staining was applied to ensure equal loading of the gels. *P < 0.05. [file Image_2.JPEG]

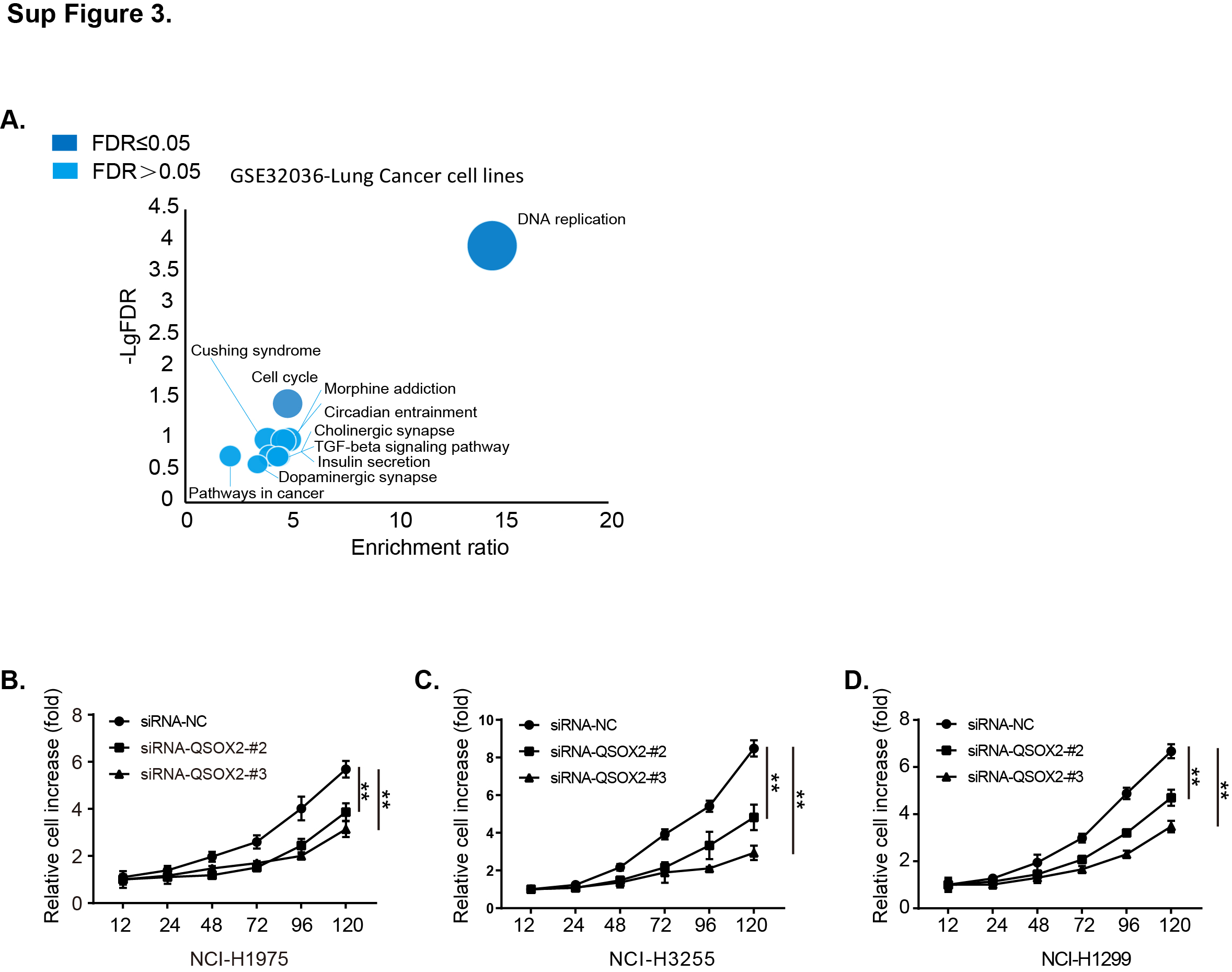

Supplement: Supplementary Figure 3 — Kyoto Encyclopedia of Genes and Genomes pathway enrichment analysis of QSOX2-related genes. (A) KEGG enrichment bubble chart. Negative log base 10 FDR values from the pathway enrichment analysis were plotted. Dark blue: FDR < 0.05; light blue: FDR ≥ 0.05. (B–D) NSCLC cells transfected with the indicated plasmids, and the cell numbers were measured at the indicated time points using flow cytometry. The data shown represent the mean ± SD, n = 3, ∗∗P < 0.01. [file Image_3.JPEG]

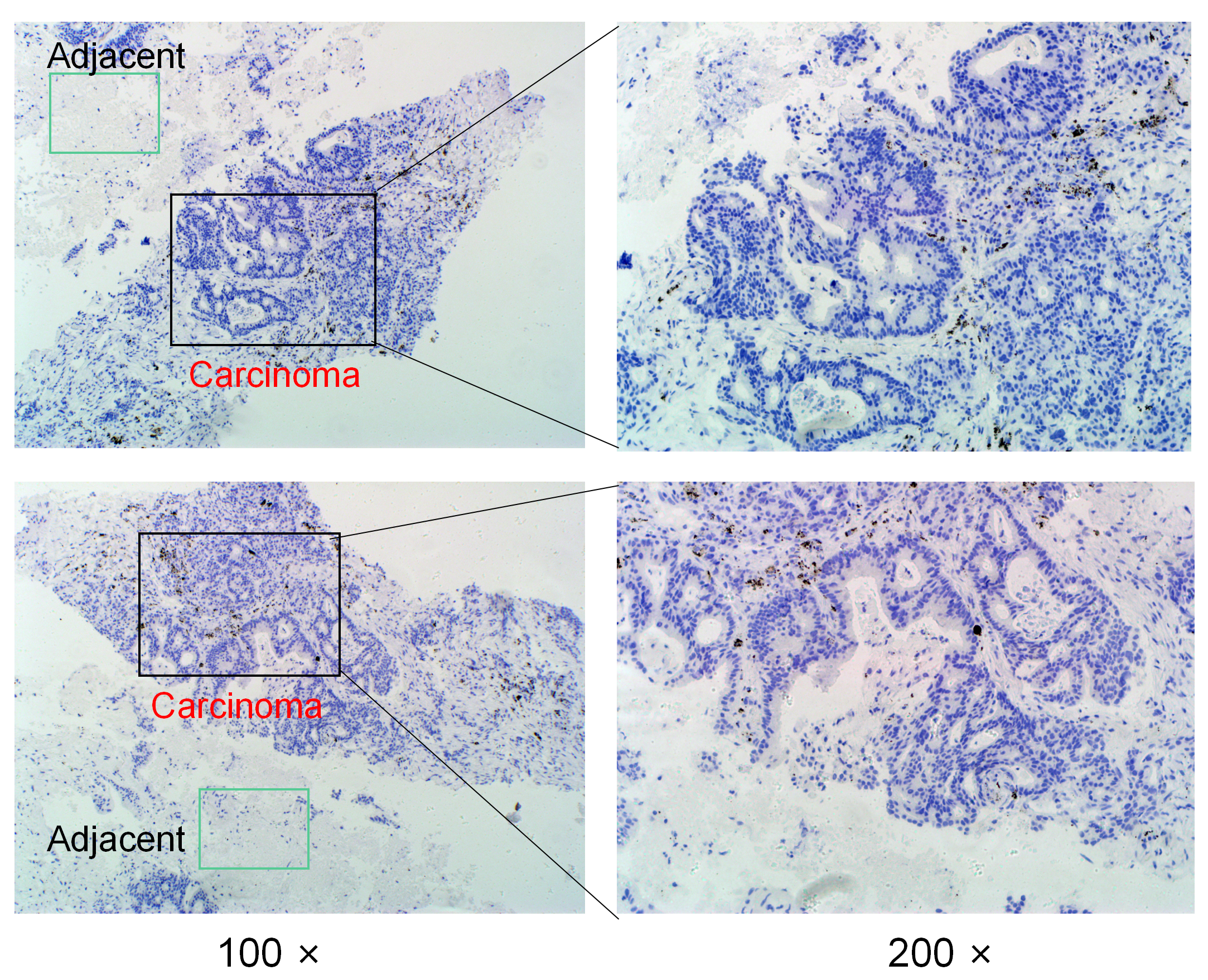

Supplement: Supplementary file 4 [file Image_4.TIF]
