# Supplementary material for: QSOX2 Is an E2F1 Target Gene and a Novel Serum Biomarker for Monitoring Tumor Growth and Predicting Survival in Advanced NSCLC
Source: Front Cell Dev Biol. 2021 Jul 19;9:688798. doi: 10.3389/fcell.2021.688798 (PMC8326667; doi:10.3389/fcell.2021.688798)
Supplement: Supplementary file 6 [file Table_2.DOCX]

**Supplementary Table 2: The siRNAs used for specific genes knockdown**

| **ID** | **Target sequences (5′-3′)** | **Start** |
| --- | --- | --- |
| siRNA-NC | sense: UUCUCCGAACGUGUCACGUTT  antisense: ACGUGACACGUUCGGAGAAT |  |
| siRNA-E2F1-#1(416) | sense: GGACCUGGAAACUGACCAUTT  antisense: AUGGUCAGUUUCCAGGUCCTT | 416 |
| siRNA-E2F1-#2(971) | sense: CUUCGGAGAACUUUCAGAUTT  antisense: AUCUGAAAGUUCUCCGAAGTT | 971 |
| siRNA-E2F1-#3(799) | sense: GACCACCUGAUGAAUAUCUTT  antisense: AGAUAUUCAUCAGGUGGUCTT | 799 |
| siRNA-QSOX2-#1(658) | sense: GCAGCCAUUACGUGGCUAUTT  antisense: AUAGCCACGUAAUGGCUGCTT | 658 |
| siRNA-QSOX2-#2(1441) | sense: GGUACGUUCACACCUUCUUTT  antisense: AAGAAGGUGUGAACGUACCTT | 1441 |
| siRNA-QSOX2-#3(1547) | sense: GCUGUGGAAGAAGCAUAAUTT  antisense:AUUAUGCUUCUUCCACAGCTT | 1547 |
