# Supplementary material for: QSOX2 Is an E2F1 Target Gene and a Novel Serum Biomarker for Monitoring Tumor Growth and Predicting Survival in Advanced NSCLC
Source: Front Cell Dev Biol. 2021 Jul 19;9:688798. doi: 10.3389/fcell.2021.688798 (PMC8326667; doi:10.3389/fcell.2021.688798)
Supplement: Supplementary file 7 [file Table_3.DOC]

**Supplementary Table 3**

**Primers used for real-time PCR amplification**

| **ID** | **Forward primer (5′-3′)** | **Reverse primer (5’-3’)** |
| --- | --- | --- |
| QSOX2 | 5’- TCCCTTCTTGACAACCGTGG-3’ | 5’- AAATGCTTTGTCCCCGTCCA-3’ |
| GAPDH | 5’- GAAGGTGAAGGTCGGAGTC-3’ | 5’-GAAGATGGTGATGGGATTTC-3’ |

**Primers used for ChIP**

| **Gene** | **Predictive BS (5’ to 3’)** | **Forward primer (5′-3′)** | **Reverse primer (5’-3’)** |
| --- | --- | --- | --- |
| QSOX2 | E2F1-BS1  5’-GGGGCGGGGAG-3’  E2F1-BS2  5’-TGGGCGGGGAG-3’ | 5’- CGTCGTGCACTTCCATTTGC-3’ | 5’- ACGTCTGACCCCGCCCTC-3’ |
