# Supplementary material for: QSOX2 Is an E2F1 Target Gene and a Novel Serum Biomarker for Monitoring Tumor Growth and Predicting Survival in Advanced NSCLC
Source: Front Cell Dev Biol. 2021 Jul 19;9:688798. doi: 10.3389/fcell.2021.688798 (PMC8326667; doi:10.3389/fcell.2021.688798)
Supplement: Supplementary file 8 [file Table_4.DOC]

**Supplementary Table 4**

**Inserted sequences of QSOX2_WT and QSOX2_MUT**

| **Name** | **Inserted sequences (5’-3’)** | **Length(bp)** | **Vector** |
| --- | --- | --- | --- |
| QSOX2_WT | 5’-ctttgagtggatgttttgctgcattttctaagcgttccaatatcatattaaaaagtcgaggctggcttgcagaccctctgaagccccaggtggggttgccaagtaaaagaaccccggaatagttttttacgtctaagaatgtcttgttaaatttgtatttcaggcatggcacagacctaggctgaaaaaccgactcactgtttgtctgaaattcacgttgaacctggcgtcgtgcacttccatttgctaaatgtggccacctggccctgttcagggaaggggtttggggaacgcgcggcggggtggggcggggcctgggcggagcctgccgcgcctgggcggggagatggcgtcatcagactcttgggggcggggctgccacgcatgggcgggggcgggggcggggagagagggcggggtcagacgtgagggggcggggcggaacagaactgggcgcgcgggggcggggccgggcgcgccggcgcacgtgacggtggttg-3’ | 500 | pGL3-Basic |
| QSOX2_MUT | 5’-ctttgagtggatgttttgctgcattttctaagcgttccaatatcatattaaaaagtcgaggctggcttgcagaccctctgaagccccaggtggggttgccaagtaaaagaaccccggaatagttttttacgtctaagaatgtcttgttaaatttgtatttcaggcatggcacagacctaggctgaaaaaccgactcactgtttgtctgaaattcacgttgaacctggcgtcgtgcacttccatttgctaaatgtggccacctggccctgttcagggaaggggtttggggaacgcgcggcggggtggggcggggcctgggcggagcctgccgcgccgggcggggccgggcgcgccggcgcacgtgacggtggttg-3’ | 374 | pGL3-Basic |
