# Supplementary material for: QSOX2 Is an E2F1 Target Gene and a Novel Serum Biomarker for Monitoring Tumor Growth and Predicting Survival in Advanced NSCLC
Source: Front Cell Dev Biol. 2021 Jul 19;9:688798. doi: 10.3389/fcell.2021.688798 (PMC8326667; doi:10.3389/fcell.2021.688798)
Supplement: Supplementary file 9 [file Table_5.DOCX]

GeNorm Analysis Results (https://seqyuan.shinyapps.io/seqyuan_prosper/)

Internal reference primers used for internal reference preferences.

| Gene Symbol | Primers (5'-3') |
| --- | --- |
| ACTB | F:GAAGATCAAGATCATTGCTCCT |
|  | R:TACTCCTGCTTGCTGATCCA |
| PPIA | F:GAAGATCAAGATCATTGCTCCT |
|  | R:TGCTGGTCTTGCCATTCCT |
| PGK1 | F:GCCACTTGCTGTGCCAAATG |
|  | R:CCCAGGAAGGACTTTACCTT |
| TPT1 | F:AAATGTTAACAAATGTGGCAATTAT |
|  | R:AACAATGCCTCCACTCCAAA |
| B2M | F:ACTGAATTCACCCCCACTGA |
|  | R:CCTCCATGATGCTGCTTACA |
| GAPDH | R: GAAGATGGTGATGGGATTTC |
|  | F: GAAGGTGAAGGTCGGAGTC |

Input table with 2^-ΔCT^


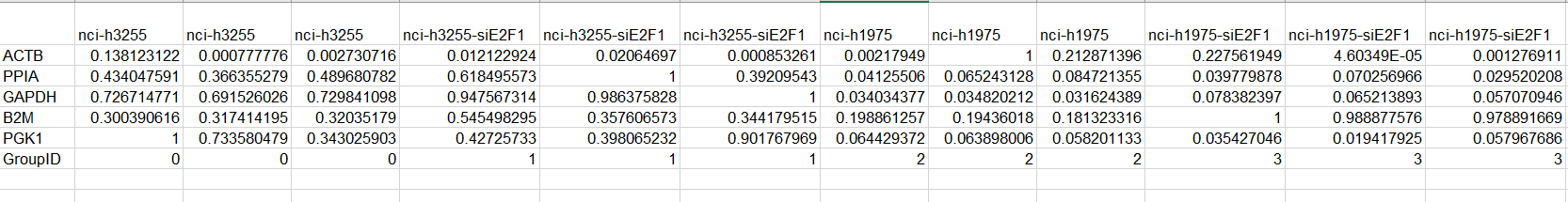


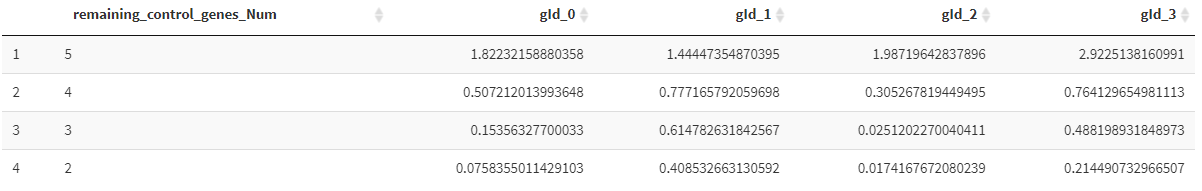


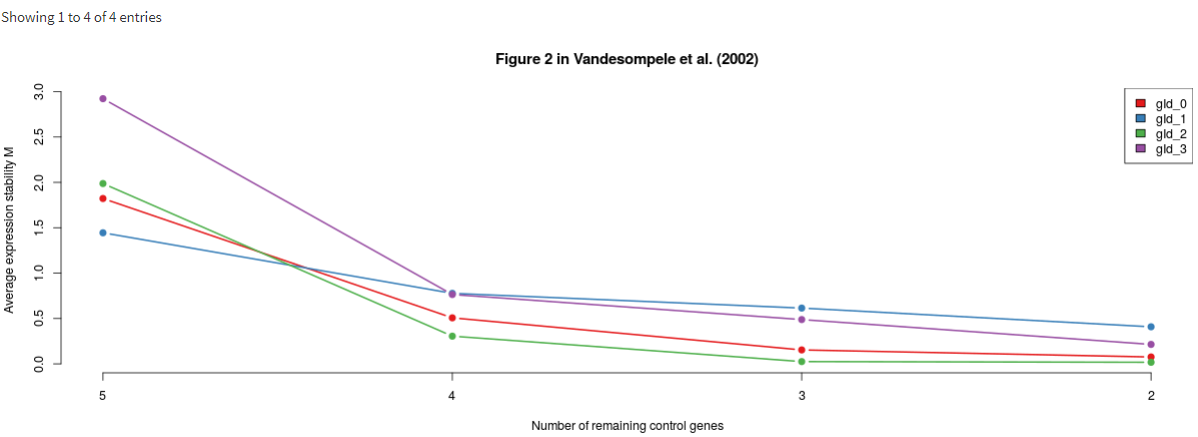


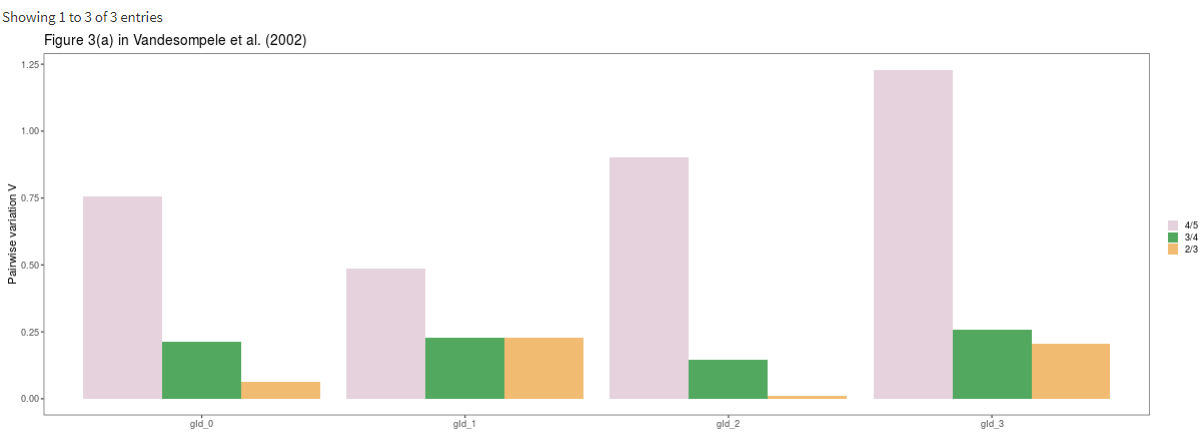

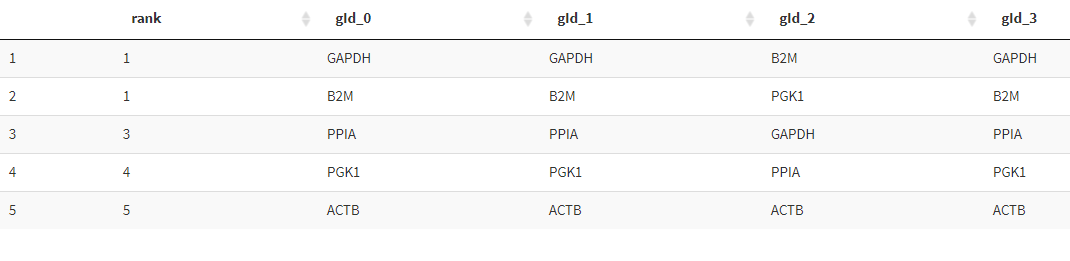


The results of GeNorm confirmed the preferential selection of GAPDH as an internal reference for non-small cell lung cancer in this study.
